# NDK-1, the Homolog of NM23-H1/H2 Regulates Cell Migration and Apoptotic Engulfment in *C. elegans*

**DOI:** 10.1371/journal.pone.0092687

**Published:** 2014-03-21

**Authors:** Luca Fancsalszky, Eszter Monostori, Zsolt Farkas, Ehsan Pourkarimi, Neda Masoudi, Balázs Hargitai, Maja Herak Bosnar, Martina Deželjin, Annamária Zsákai, Tibor Vellai, Anil Mehta, Krisztina Takács-Vellai

**Affiliations:** 1 Department of Genetics, Eötvös Loránd University, Budapest, Hungary; 2 Laboratory for Molecular Oncology, Division of Molecular Medicine, Rudjer Bošković Institute, Zagreb, Croatia; 3 Department of Biological Anthropology, Eötvös Loránd University, Budapest, Hungary; 4 Medical Research Institute, Ninewells Hospital Medical School, University of Dundee, Dundee, United Kingdom; CSIR-Central Drug Research Institute, India

## Abstract

Abnormal regulation of cell migration and altered rearrangement of cytoskeleton are characteristic of metastatic cells. The first described suppressor of metastatic processes is NM23-H1, which displays NDPK (nucleoside-diphosphate kinase) activity. To better understand the role of *nm23* genes in cell migration, we investigated the function of NDK-1, the sole *Caenorhabditis elegans* homolog of group I NDPKs in distal tip cell (DTC) migration. Dorsal phase of DTC migration is regulated by integrin mediated signaling. We find that *ndk-1* loss of function mutants show defects in this phase. Epistasis analysis using mutants of the α-integrin *ina-1* and the downstream functioning motility-promoting signaling module (referred to as CED-10 pathway) placed NDK-1 downstream of CED-10/Rac. As DTC migration and engulfment of apoptotic corpses are analogous processes, both partially regulated by the CED-10 pathway, we investigated defects of apoptosis in *ndk-1* mutants. Embryos and germ cells defective for NDK-1 showed an accumulation of apoptotic cell corpses. Furthermore, NDK-1::GFP is expressed in gonadal sheath cells, specialized cells for engulfment and clearence of apoptotic corpses in germ line, which indicates a role for NDK-1 in apoptotic corpse removal. In addition to the CED-10 pathway, engulfment in the worm is also mediated by the CED-1 pathway. *abl-1/Abl* and *abi-1/Abi*, which function in parallel to both CED-10/CED-1 pathways, also regulate engulfment and DTC migration. *ndk-1*(-);*abi-1*(-) double mutant embryos display an additive phenotype (e. g. enhanced number of apoptotic corpses) which suggests that *ndk-1* acts in parallel to *abi-1*. Corpse number in *ndk-1*(-);*ced-10*(-) double mutants, however, is similar to *ced-10*(-) single mutants, suggesting that *ndk-1* acts downstream of *ced-10* during engulfment. In addition, NDK-1 shows a genetic interaction with DYN-1/dynamin, a downstream component of the CED-1 pathway. In summary, we propose that NDK-1/NDPK might represent a converging point of CED-10 and CED-1 pathways in the process of cytoskeleton rearrangement.

## Introduction

The human *nm23* (*nme*) gene family consists of ten members named after the first identified metastasis suppressor *nm23-H1* (*non metastatic clone 23*). The metastasis suppressor function has been extensively corroborated using metastatic cell lines (melanoma, breast-, colon-, hepato- and oral squamous cell carcinoma) where, for the most part, overexpression of NM23-H1 was associated with reduced cell motility (reviewed in [1]). Proteins encoded by the *nm23* family are classified into two groups. Isoforms of group I (NM23-H1–NM23-H4) possess nucleoside diphosphate kinase activity and are highly conserved in eukaryotes from yeast to mammals [2]. Beyond their nucleoside diphosphate kinase activity, additional molecular functions are associated with NDPKs such as histidine-dependent protein kinase activity [3]–[4], 3′-5′ exonuclease action [5]–[6], DNase activity in caspase-independent apoptosis [7] and transcriptional regulation [8]. Together, group I members display essential functions; both up- and down-regulation can disrupt growth and/or differentiation [9]; [10].

The most extensive set of studies analyzing group I members' role in cell motility and migration have utilized *Drosophila. awd*, the fly orthologue of *nm23-H1/H2* is a negative regulator of migrating tracheal and border cells via modulating endocytosis of different receptors, such as platelet-derived growth factor receptor (PDGFR)/vascular endothelial growth factor receptor (VEGFR) [11] and fibroblast growth factor receptor (FGFR) [12]. In the process which affects the level of FGFRs Awd functions together with the dynamin/Shibire in endocytosis as a putative GTP supplier for the GTPase [9]. Although no physical association of Awd and Shibire could be demonstrated in *Drosophila*, in rat a direct interaction was detected between NDPK and dynamin I by *in vitro* pulldown and coimmunoprecipitation [13]. Independent studies using *Dictyostelium* also confirm links to light-dependent, vectorial cell migration and cell nutrition through different forms of endocytosis [10].

*Caenorhabditis elegans* serves as a particularly amenable model to investigate the process of cell migration. The nematodes are transparent and have simple anatomy making it possible to follow the migration of individual cells in the living animal throughout development. Well studied migrating cell types of *C. elegans* include sex myoblasts (SM), two Q neuroblasts (QL and QR) and their descendants, and distal tip cells (DTCs) or the gonadal leader cells [14]–[16]. In *C. elegans*, we identified and described a single group I NDPK ortholog, NDK-1, which shows 85% and 86% similarity to NM23-H1 and H2, respectively [17]. In the current study we examine the role of NDK-1 in DTC migration and apoptosis.

**Figure 1:**
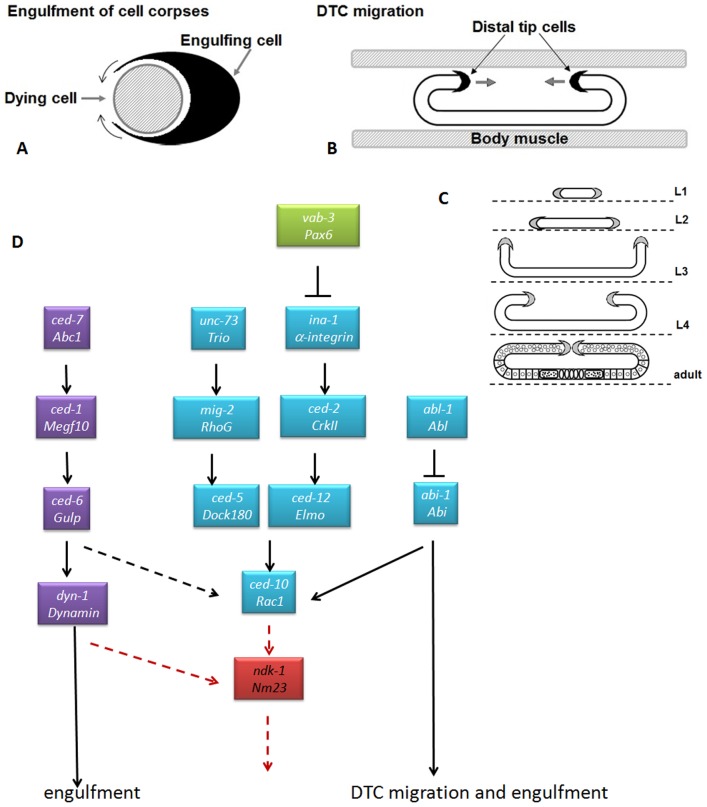
Site of action of NDK-1 in the analogous processes of engulfment and DTC migration. **A, B**: Cell corpse engulfment and DTC migration are similar processes. In each case, the surface membrane of a cell (black) extends along the surface of another cell (hatched). The small arrows near the black cells indicate the directions of cell-surface extension. **B**: Only the relevant parts of body muscles are shown [19]. **C**: Schematic review of DTC migration (based on [21]). DTCs are located on the distal edges of the gonad primordium and start to migrate in L2. They migrate along the ventral surface (dashed line) of the hermaphrodite in L2 (first or ventral phase). Then they turn to the dorsal side during L3 (second or ventral to dorsal phase). A second turn redirects migration along the dorsal surface toward the center of the nematode during L4 (third or dorsal phase). The end of the migration is dorsal to the vulva, resulting in the mirror image U-shaped gonad of the adult. The developmental stage is indicated at right of each diagram. **D**: Signaling pathways in engulfment and DTC migration (based on [21]–[22]. Common genes are blue, green colour indicates the factors involved only in DTC migration, genes in purple boxes play a role only in engulfment. We suggest that NDK-1/NM23 acts downstream of CED-10/Rac in the processes of DTC migration and engulfment of apoptotic corpses. NDK-1 shows a genetic interaction with DYN-1/Dynamin.

The tightly regulated migratory path of DTCs provides an elegant system to explore how cell migration can be guided within the spatial and temporal context of the organism. Distal tip cells are specialized leader cells and are responsible for gonad morphogenesis via their migration in *C. elegans* [16]. During the four stages of larval development (L1, L2, L3, and L4), DTCs migrate in response to attractive and repulsive cues to properly form two mirror image U-shaped gonad arms (***Figure 1C***). During development, somatic cells dying by apoptosis are engulfed by the neighboring cells as there are no specialized engulfing cells. However, during germ cell death, which occurs as a part of the oogenesis program, at least half of all oogenic germ cells [17] are eliminated by apoptosis and are engulfed by gonadal sheath cells which surround the germ cells [18].

DTC migration and engulfment of apoptotic corpses are analogous processes in *C. elegans* (***Figure 1***, [19]–[22]). Both require cytoskeletal rearrangements and membrane trafficking/recruitment (***Figure 1A, B***) sharing genes regulating both processes (***Figure 1D***, [22]–[23]). Two major partially redundant pathways regulate engulfment. The CED-10/Rac pathway consists of *unc-73/Trio*, *ced-2/CrkII, mig-2/RhoG, ced-5/DOCK180*, *ced-12/ELMO* and *ced-10/Rac* genes acting downstream of the alpha integrin receptor *ina-1*, while the CED-1 pathway includes *ced-7/ABC1*, the *ced-1/MEGF10* receptor, *ced-6/GULP*, and *dyn-1/Dynamin* [22]–[24]. The CED-10/Rac pathway controls both engulfment and the movement of DTCs by rearrangeing the cytoskeleton of the engulfing and migrating cells. The CED-1 pathway is involved only in engulfment where it recruits membranes to extend the surface of the engulfing cell. It has been suggested that a third, distinct pathway consisiting of *abl-1* and *abi-1* also influences both DTC migration and engulfment in parallel to the CED-10 Rac and CED-1 pathways (***Figure 1D***) [*22*].

In this study we demonstrate that NDK-1 is required for normal DTC migration and engulfment of apoptotic corpses. We show that *ndk-1* influences both processes via common genes, acts downstream of *ced-10* (cell death abnormality)/*Rac* and in parallel to *abi-1* (Abl interactor)/*ABI*; and additionally shows a genetic interaction with *dyn-1/dynamin*. Thus, NDK-1 affects the rearrangement of cytoskeleton in both DTC migration and apoptotic engulfment. We also show that NDK-1 functions similar to its human counterparts in cell migration, as it inhibits the migratory potential of invasive breast adenocarcinoma cells. Our results might help to better understand the function of *nm23* genes in metastasis.

## Results

### *C. elegans* FLAG::NDK-1 reduced the motility of MDA-MB-231T cells

**Figure 2:**
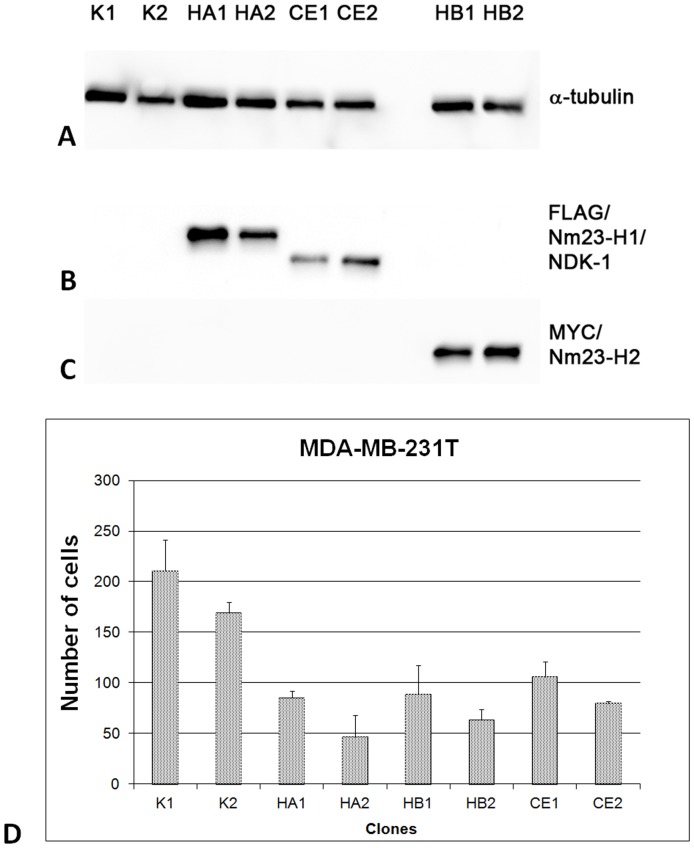
Western blot analysis and migration assay of transfected MDA-MB-231T cells. MDA-MB-231T cells were stably transfected with, pcDNA3 (K1 and K2), pcDNA3/FLAG-*nm23-H1* (HA1 and HA2), pcDNA3/FLAG-*ndk-1* (CE1 and CE2) and pcDNA3/MYC-*nm23-H2* (HB1 and HB2). **A**: Western blot with anti-α-tubulin antibodies (loading control). **B**: Western blot with anti-FLAG- antibodies, visible band in HA1, HA2, CE1 and CE2 proves stable overexpression of introduced transgenes. **C**: Western blot with anti-MYC- antibodies, visible band in HB1 and HB2 (overexpression of NM23-H2). **D**: Migration assay. MDA-MB-231T cells stably transfected with one of the following constructs: pcDNA3 (K1 and K2), pcDNA3FLAG/*nm23*-H1 (HA1 and HA2), pcDNA3FLAG/*ndk-1* (CE1 and CE2) and pcDNA3/MYC-*nm23-H2* (HB1 and HB2) were tested for migration potential. The cells were stained with crystal violet and counted (the number of migrated cells were counted in four representative microscopic fields per each clone). The CE1 and CE2 clones as well as HA1, HA2, HB1 and HB2 exhibited significantly diminished migration potential compared to control (K1 and K2) clones (Student's t-test, p<0.05). The results are presented as an absolute number of migrated cells in 4 representative fields for every clone (±SD).

Our group is investigating the function of nucleoside diphosphate kinases (NDPKs) in the model organism *C. elegans*. NDK-1 is the single group I NDPK homolog of the worm and shows high sequence similarity to NM23-H1 and H2 [25]. It is known that *nm23* genes regulate cell migration [26]. For example overexpression of NM23-H1 and its sponge ortholog both reduced the migratory and invasive potential of CAL27 (oral squamous carcinoma of the tongue) cells [27]. Based on the high sequence similarity one might expect that the *C. elegans* homolog of NM23-H1/H2 is also able to act likewise. Therefore we investigated the effect of NDK-1 exerted on the cell migration capacity of the breast adenocarcinoma MDA-MB-231T cell line. MDA-MB-231T cells are far more migratory than CAL27 cells, and the influence of NM23-H1 is much more obvious in these cells. Stably transfected MDA-MB-231T cells overexpressing FLAG::NDK-1, FLAG::NM23-H1 and MYC-NM23-H2 (***Figure 2A,B,C***) were used for migration assay.Three independent experiments demonstrated that NDK-1 clones CE1 and CE2 both diminished the migratory potential of MDA-MB-231T cells in a similar manner to overexpressing NM23-H1 (clones HA1 and HA2) or NM23-H2 (HB1 and HB2) clones (***Figure 2D***). The suppression of migratory potential reached or exceeded 50% in almost all clones overexpressing either the worm or a human NM23 homolog compared to MDA-MB-231T control clones (clones K1 and K2).

### *ndk-1*(*lf*) mutants show incomplete migration of DTCs

We recently characterized the worm ortholog of group I NDPKs, *ndk-1*, and identified the strong loss of function allele *ok314*. *ok314* is a 1157 bp-long deletion, which removes the entire *ndk-1* ORF, as well as upstream and downstream regulatory sequences [25].

**Figure 3:**
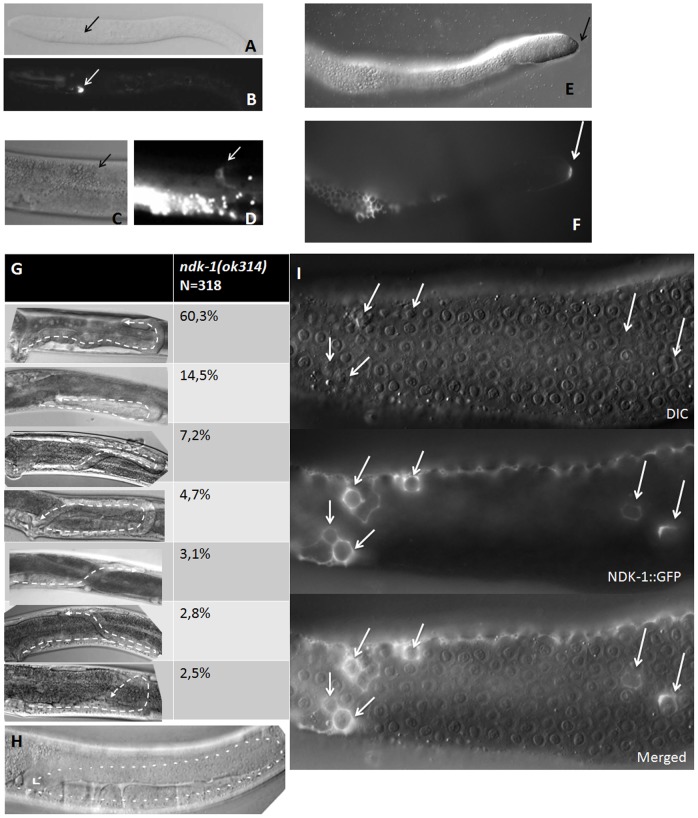
NDK-1 functions in DTCs. NDK-1::GFP shows expression in distal tip cells (DTCs) in TTV2 (**B, D**) and TTV3 (**F**) translational reporter lines in L3 (**A, B**), L4 (**C, D**) larvae and adults (**E, F and I**). **A**, **C**, and **E** are the corresponding DIC images of **B**, **D**, and **F**, respectively. DTC locations are indicated with arrows. We note that on panel **D** GFP expression in DTC (marked by an arrow) is overshadowed by intense expression of autofluorescent granules in the intestine. **E, F and I**: gonads were isolated from adult animals. **H**: DIC image of an adult wild-type (N2) gonad arm. **G**: Variation and distribution of DTC migration phenotypes observed in *ndk-1*(*ok314*) mutants (see details in the main text). The migratory path of DTCs is marked by dashed lines. **I**: In TTV3 lines carrying the integrated transgene, NDK-1::GFP expression is also observed in gonadal sheath cells. DIC, fluorescent and merged images derive from the gonadal loop region of a transgenic animal. On the DIC panel white arrows show dying cells, which are surrounded by sheath cells strongly expressing NDK-1::GFP (white arrows on the fluorescent and merged panels).

*ndk-1*(*ok314*) mutants show a Pvl (protruding vulva) phenotype and they are sterile due to germ cell arrest in mitotic phase [25]. Morphological studies using Nomarski optics revealed that *ndk-1*(*ok314*) animals have insufficiently elongated gonad arms suggesting defects in DTC migration. Detailed analysis (***Figure 3G,H***) of 318 gonad arms showed that in the majority of *ok314* mutants (60.3%) DTCs turned to the proper direction and side however their migration is stopped prior to reaching the vulva. In 14.5% of the cases DTCs turned back to the vulva but in the ventral (instead of the dorsal) side or initiated ventral migration but subsequently vectored to the dorsal side before the turn (7.2%). Other defects manifested low penetrance (wrong direction, lack of the turn, wandering, bizzare twists) and we observed normal DTC migration in only 4.7% of the animals. Altogether we conclude that the prominent phenotype of *ok314* mutants is incomplete migration of DTCs.

In order to determine whether NDK-1 is expressed in DTCs, we studied NDK-1 expression in transgenic strains TTV2 and TTV3. Both were generated using the same translational construct but by distinct methods. In case of TTV2, the strain was created by ballistic bombardment, which resulted in extrachromosomal arrays of the transgene and therefore genetic mosaicism. For TTV3 the MosSCI method was applied to integrate a single copy of the transgene into the genome. The latter method allowed the rescue of Ndk-1 mutant phenotypes. Among TTV2 and TTV3 animals we observed expression in DTCs from L3 (***Figure 3 A, B***) to L4 (***Figure 3C,D***) stage while in the isolated gonads of TTV3 animals DTC expression was also detected in adults (***Figure 3E,F***).

As *ndk-1* is expressed in the distal tip cells in different developmental stages and knockout of *ndk-1* results in different DTC migration defects (***Figure 3G,H***), we hypothesized that *ndk-1* functions in the process of DTC migration.

### *ndk-1*(-) reduces the extra turn phenotype of CED-10 pathway mutants while *abi-1* restores the Ndk-1 DTC migration phenotype

Integrins are heterodimeric receptors consisting one α and one β subunit. They provide connections between the extracellular matrix and the actin cytoskeleton regulating cellular shape, motility and cell cycle. Integrin mediated signaling is well known to be involved in the process of DTC migration [21]. Interestingly, both NM23-H1 and H2 have been linked to integrins [28]–[30].

The *C. elegans* genome encodes two α (*ina-1*: integrin alpha, *pat-2*: paralysed arrest two-fold embryo stage) and one β (*pat-3*) subunits [21]. All these genes are expressed and function in DTCs [21]; [31]–[32]. Genes encoding *C. elegans* integrins are essential, null mutations in any of them cause embryonic lethality [21], [31]. Since hypomorph integrin mutants are viable, we applied these mutant alleles for DTC analysis. Hypomorph *ina-1* mutants show an abnormal migration (i. e. the migratory path of DTCs is often longer compared to wild-type and extra turns occur due to pathfinding defects, see also definition in methods) phenotype because DTCs do not stop prior to reproduction [21]. Silencing of *pat-2* by RNAi resulted in dorsal pathfinding defects [21]. An incomplete migration with an enlarged and blunt end of the gonad arm can be observed with DTC-specific expression of a dominant-negative *pat-3* transgene or by *pat-3*(*RNAi*) [21], [31]–[32].

*vab-3* (variable abnormal morphology) encodes a homeodomain protein, the *C. elegans* orthologue of Pax6. *vab-3/Pax6* transcriptionally regulates both α-integrins in different manners: it downregulates *ina-1* (***Figure 1D***) to cause the cessation of DTC migration and activates *pat-2* expression at L3 stage which is necessary for normal dorsal pathfinding [21]. *vab-3* reduction of function mutants show an overmigration phenotype similar to *ina-1*(*rf*) mutants.

The Rac GTPases CED-10 and MIG-2 (abnormal cell migration) have been shown to act downstream of INA-1 during the migration of DTCs [21]. Additionally, *ced-10* was found to function together with *ced-2* and *ced-5* in DTC migration (reviewed in [16]). Moreover, in the last decade, an entire pathway (often called as CED-10 pathway) was built using also the above mentioned genes (***Figure 1D***). [19], [21]–[22]. Mutations in CED-10 pathway components manifest characteristically extra turns due to pathfinding defects. Furthermore, these genes – similar to integrin signaling components – function during the dorsal (or third) phase (***Figure 1C***) of DTC migration [16].

**Figure 4:**
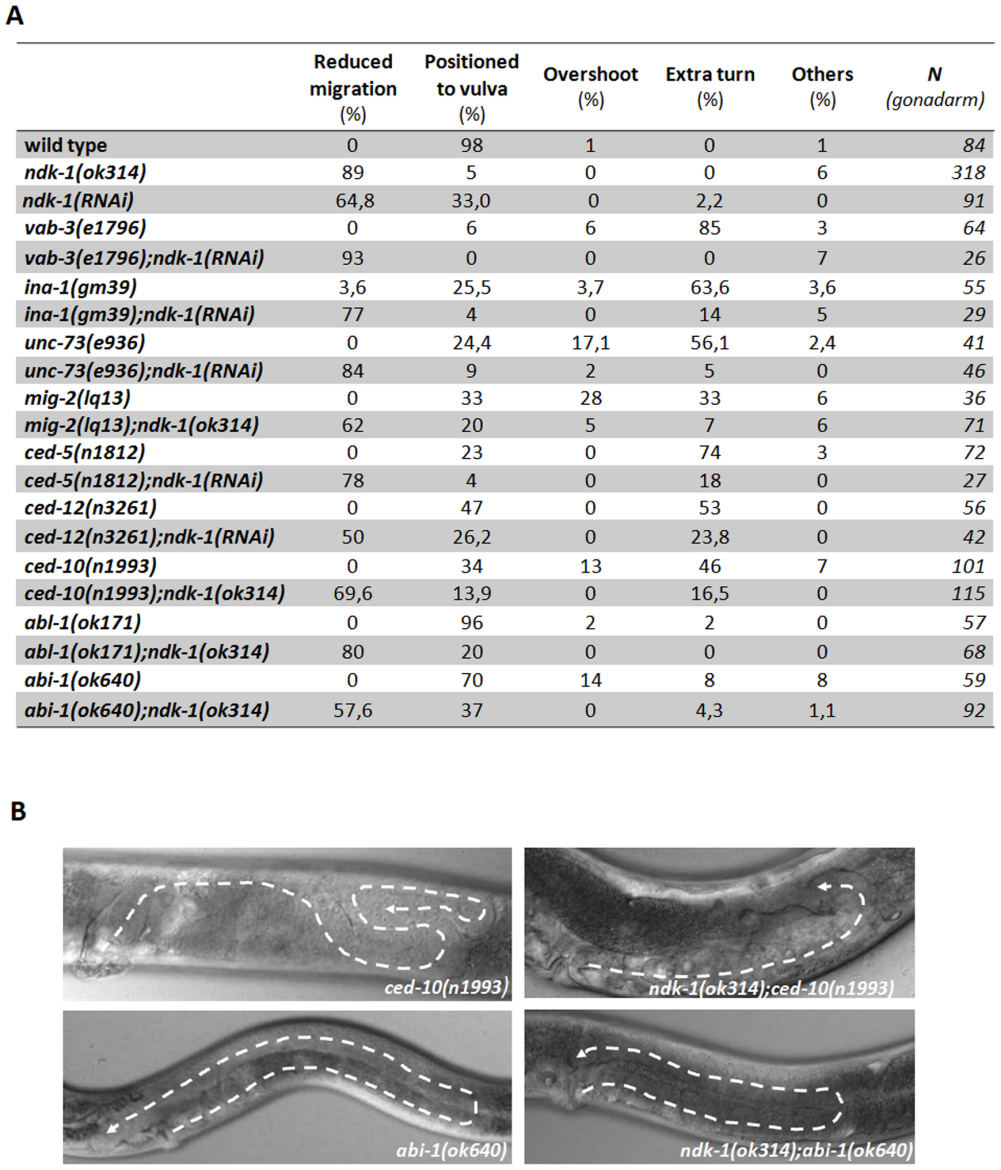
*ndk-1* functions likely downstream of *ced-10* in the process of DTC migration and shows a genetic interaction with *abi-1*. A: Epistasis analysis using mutants of the homeodomain transcription factor *vab-3*, the α-integrin *ina-1* and the downstream functioning CED-10 pathway genes suggests that NDK-1 acts downstream of CED-10/Rac. Loss of *ndk-1* reduced the extra turn phenotype of *vab-3, ina-1, unc-73, mig-2*, *ced-5, ced-12* and *ced-10* mutants (B) and mostly resulted in Ndk-1-like gonad (B). Examining double mutants of *ndk-1* and the parallel pathway functioning in DTC migration, we found that *abl-1*(*ok171*) did not influence the reduced migration of *ndk-1* mutants. However, the *abi-1*(*ok640*) mutation (B) was able to partially restore the DTC migration defects of *ndk-1*(*ok314*) animals (B). The different DTC migration categories are described in Materials and Methods. Statistical analysis used Fisher's exact test. The difference in the distribution of migration phenotypes was significant (Fisher's exact test, p≤0.05) in all pairs of single and double mutants (with the exception of *ndk-1*(*RNAi*) and *vab-3*(*e1796*);*ndk-1*(*RNAi*) mutants (p>0.05)).

ABL-1 and ABI-1 function in a distinct, recently discovered cascade, acting parallel to the CED-10 pathway in DTC migration (***Figure 1D***, [22]). *abl-1*(*ok171*) and *abl-1*(*n1963*) alleles manifest no obvious gonadal phenotype [22] but the DTC migration defects of *ced-2*, *ced-5, ced-12* and *ced-10* mutants were suppressed in *abl-1* mutant backgrounds. ABL-1 inhibits ABI-1 in *C. elegans*, *abi-1*(*tm494*) mutation itself or *abi-1*(*RNAi*) caused only weak defects in DTC migration [22], however *abi-1*(*RNAi*) enhanced the extra turns of *ced-5*(*n1812*) and *ced-12*(*n3261*) [22]. We analyzed DTC migration in *abi-1*(*ok640*) mutants and observed 26% of abnormal gonad arms in which the overshoot phenotype was the most frequent (13%) (***Figure 4 A, B***).

NDK-1 expression was detected in DTCs from L3 larval stage onwards, and lack of NDK-1 activity resulted in early cessation of migration in the dorsal phase in most of the cases (***Figure 3G***). Based on these facts we investigated the connection between *ndk-1* and integrin signaling genes during the process of DTC migration.

We performed an epistasis analysis (a genetic tool suitable for ordering genes into pathways) using the *ok314* mutation or RNAi mediated silencing of *ndk-1* and the following loss of function or reduction of function mutants: *vab-3(e1796), ina-1(gm39), ced-10(n1993), mig-2(lq13), unc-73(e936), ced-5(n1812), ced-12(n3261), abl-1(ok171)*/ABL1, *abi-1*(*ok640*). Except *abl-1*(*ok171*) and *abi-1*(*ok640*), all these mutants display large gonads with extra turns (***Figure 4A***).

To examine the effect of the absence of NDK-1 in the above mentioned mutant backgrounds, we either depleted the function of NDK-1 by RNAi or generated double mutants. In the cases of *vab-3(e1796), ina-1(gm39), unc-73(e936), ced-5(n1812)* and *ced-12*(*n3261*) RNAi was applied to knockdown NDK-1 function in the corresponding mutant background. In the cases of *mig-2(lq13), ced-10(n1993), abl-1(ok171)* and *abi-1*(*ok640*) double mutants were created by introducing the *ok314* mutant allele of *ndk-1* into the appropriate single mutants.

Loss of *ndk-1* suppressed the extra turns of *vab-3, ina-1, unc-73, mig-2*, *ced-5, ced-12* and *ced-10* mutants and mostly resulted in Ndk-1-like gonad (reduced gonad with fewer extra turns) (***Figure 4 A, B***). Although, as mentioned above, *abl-1*(*ok171*) was able to suppress the phenotype of the *ced* mutants [22], it did not influence the reduced migration of *ndk-1* mutants. Unlike *abl-1*(*ok171*), *abi-1*(*ok640*) mutation was able to restore the DTC migration defects of *ndk-1*(*ok314*) animals in 37% of the cases (***Figure 4 A, B***), suggesting that *ndk-1* might either act upstream of *abi-1* or loss of ABI-1 compensates for the absence of NDK-1, indicating parallel functions of the two genes.

Based on these results we suggest that *ndk-1* might act downstream of or in parallel to *ced-10/Rac* and upstream of or in parallel to *abi-1/Abi* (***Figure 1D***) in the process of DTC migration. Our data raise the possibility that *ndk-1* acts downstream of *ced-10* and in parallel to *abi-1*. Although this tendency could be hypothesized on the basis of the distribution of migration phenotypes, it could not be confirmed by statistical analyses (***Figure 4A***). Thus, the epistatic relationship of NDK-1 and the two parallel pathways cannot be deduced unambiguously, as DTC migration phenotypes are difficult to quantify. To specify NDK-1's site of action more precisely, we decided to analyze the role of NDK-1 in apoptosis, where phenotypes, (e.g. number of apoptotic corpses, so called Ced phenotype) can be quantified with precision.

### *ndk-1*(-);*abi-1*(-) double mutants show an additive Ced phenotype, suggesting that NDK-1 acts downstream of CED-10, in parallel to ABI-1

Since CED-10/Rac signaling controls both DTC migration and engulfment, and our data derived from the DTC analysis suggested that NDK-1 acts downstream of or in parallel to CED-10, we hypothesized that NDK-1 also functions in the engulfment phase of apoptotis. In somatic tissues of the worm, elimination of apoptotic corpses occurs by the non-specialized neighbouring cells, however cell corpses in the germline are all engulfed by gonadal sheath cells [17]–[18]. Transgenic worms of the strain TTV3 show that NDK-1::GFP is expressed in these somatic gonadal cells around dying germ cells (***Figure 3I***), which further supports the potential role of NDK-1 in engulfment. To address this issue, first we examined the number of germ cell corpses in *ndk-1* mutants. Among germ cells, only the developing oocytes die via apoptosis when they exit the pachytene phase at the gonadal loop region [17]–[18].

**Figure 5:**
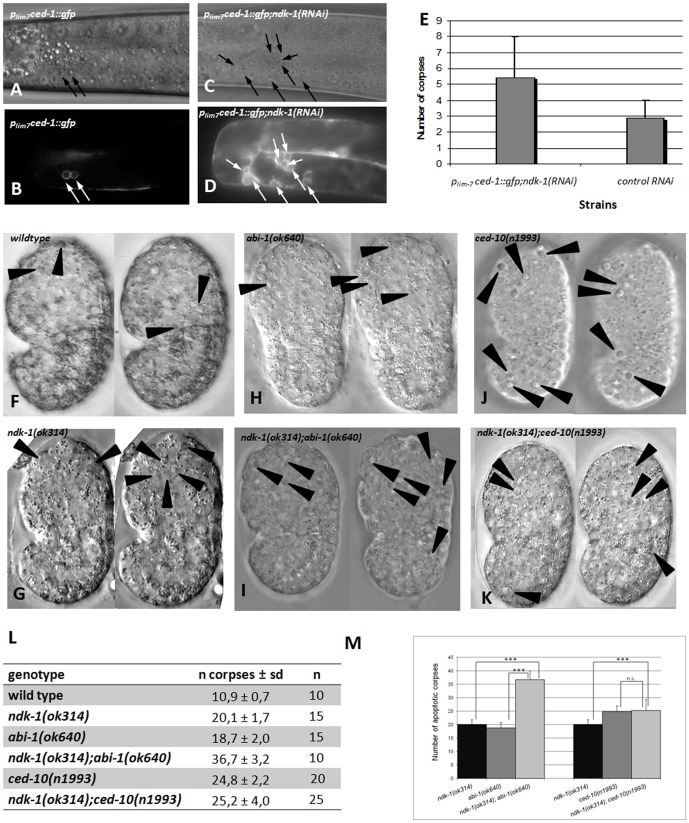
*ndk-1*(*ok314*);*abi-1*(*ok640*) double mutants show additive Ced phenotye. *P_lim-7_ced-1::gfp* transgenic worms treated by *ndk-1*(*RNAi*) (**D**) show an excess of apoptotic corpses in the germline compared to worms carrying the same transgene treated by control RNAi (**B**). **A**, **C** are corresponding DIC images of **B**, **D** respectively. Arrows indicate apoptotic germ cells. **E**: Increase of apoptotic germ cell death in *ndk-1*(*RNAi*) animals compared to the control, where p*_lim-7_ced-1::gfp* transgenic worms were grown on control RNAi (e.g. *E. coli* HT115(DE3) carrying an empty vector). **F–K**: Monitoring apoptotic corpses in wild-type embryos (**F**), *ndk-1*(-) (**G**), *abi-1*(-) (**H**), *ced-10*(-) (**J**) single mutants and *ndk-1*(-);*abi-1*(-) (**I**), *ndk-1*(-);*ced-10*(-) (**K**) double mutants using DIC optics. Embryos slightly before or at the comma stage were scored. Each panel shows two focal planes (**F–K**). Arrowheads indicate apoptotic corpses. Panel **L** shows a summary of apoptotic corpses scored in *ndk-1*(*ok314*), *abi-1*(*ok640*), *ced-10*(*n1993*) single mutant and *ndk-1(ok314);abi-1(ok640)* and *ndk-1(ok314);ced-10(n1993)* double mutant embryos. *ndk-1*(-);*abi-1*(-) double mutants display an enhanced Ced phenotype compared to single mutants, however *ndk-1*(-);*ced-10*(-) doubles are reminiscent of *ced-10*(-) single mutants. Panel **M** is the graphic representation of panel **L**. *p*-values refer to comparisons of apoptotic cell corpse numbers between single and double engulfment mutants. * * * means *p*<0.001; n.s. means not significant (*p* = 0.627).

We used RNAi because germ cells of *ndk-1*(*ok314*) loss of function mutants arrest at the mitotic phase before reaching pachytene (our unpublished data), thus engulfment defects cannot be investigated in this mutant background. We analyzed the gonads of *ndk-1*(*RNAi*) worms using DIC optics and also marked the early apoptotic corpses with CED-1::GFP [24]. We observed an excessive level of GFP-labeled apoptotic corpses in *ndk-1*(*RNAi*) treated worms (6.3; N = 87) compared to the control animals (2.9; N = 65) (***Figure 5A–E***).

During *C. elegans* development, 131 somatic cells are eliminated by apoptosis [33] and most of them (109) die during mid-embyrogenesis [34]. Expecting that the lack of NDK-1 results in an accumulation of cell corpses during embryogenesis as well, we counted apoptotic corpses by DIC optics in *ndk-1*(*ok314*) and in wild type embryos around the comma stage. We observed a near doubling of corpses in *ndk-1* mutant embryos (20.1) compared to wild type (10.9), indicating that NDK-1 plays a role in apoptosis (***Figure 5F,G,L*** ***and panels A,D in Figure S1***).

Next, we examined NDK-1's function in the engulfment process during embryogenesis. We analyzed apoptotic corpses in comma stage embryos and in embryos slightly after comma stage (close to 1.5 fold stage) harboring mutations in *ndk-1* and the key positioned genes *abi-1* and *ced-10* (downstream components of the CED-10/Rac and ABL-1/ABI-1 parallel pathways). First we counted apoptotic corpses in the homozygous mutant progeny of *ndk-1(ok314)/+;ced-10(n1993)* animals and found that double mutant comma stage embryos contained on average 25.2 apoptotic corpses (***Figure 5 J, G, K, L, M*** ***and panels A,C,F in Figure S1***) which is comparable with *ced-10*(*n1993*) single mutants; 24.8. The majority of the 25 examined *ndk-1*(*ok314*);*ced-10*(*n1993*) double mutant embryos displayed cell corpses between 22–27, reminiscent of *ced-10*(-) single mutants, however around 10% of the doubles showed an increased number of cell corpses (e. g. in two embryos 38 corpses were counted) (***Figure 5 L, M***). We note that for analysing the genetic interaction between *ced-10* and *ndk-1*, the *ced-10* allele *n1993* was used, which is considered as a partial loss-of-function allele, not a null, because *ced-10* null mutations are maternal effect lethal [35].

Next, we examined the genetic interaction of NDK-1 and ABI-1 in apoptosis. *abi-1*(*tm494*) or *abi-1*(*RNAi*) alone caused only weak defects in engulfment [22]. Consistently, we detected slightly more apoptotic corpses in *abi-1*(*ok640*) embryos (18.7) compared to wild type (10.9) (***Figure 5 F, H, L, M***).

Analyzing the homozygous progeny of *ndk-1(ok314)/+;abi-1(ok640)* heterozygotes revealed that *ndk-1*(*ok314*);*abi-1*(*ok640*) double mutants displayed 36.7 cell corpses on average (***Figure 5 F, G, H, I, L, M***, ***panels B,D,E in Figure S1***). Thus, introducing *abi-1*(*ok640*) mutation into *ndk-1*(*ok314*) background we noticed an additive phenotype, an increased level of apoptotic corpses (***Figure 5 I, L, M***). This additive phenotype suggests that *ndk-1* acts in parallel to *abi-1*. Namely, if two engulfment genes act in the same linear pathway, the phenotype of the double mutants (number of corpses) should not be more severe than that of the stronger single (null) mutant. If the two genes function in parallel pathways a (null) mutation in one of them should enhance significantly the phenotype caused by the other, resulted in an additive phenotype [24], [36]. Therefore our results suggest that in the process of engulfment *ndk-1* acts in parallel to *abi-1* and downstream of *ced-10* (***Figure 1D***).

### *ndk-1*(*ok314*) embryos show a phenotype characteristic for *dyn-1*(-) mutants: late embryonic lethality with persistent cell corpses

NDPK/AWD is known as a potential GTP supplier of the large GTPase, Dynamin/Shibire in several model systems [11]–[13], [37]. The *C. elegans* ortholog of *dynamin/shibire*, *dyn-1* acts in the CED-1 engulfment pathway downstream of *ced-6* ([36]; ***Figure 1D***). DYN-1 is proposed to organize vesicle transport: 1. to the phagocytic cups for extending pseudopods during engulfment and 2. to phagosomes for apoptotic cell degradation [36].

*dyn-1* loss of function mutants are embryonic lethal [36], [38] due to endocytosis defects. Beyond this main phenotype, defective DYN-1 causes failure in engulfment [36]. Many *dyn-1* mutant alleles bearing missense mutations in the GTPase domain result in embryonic lethality at later embryonic stages and a strong Ced phenotype. These embryos do not move inside the eggshell in contrast to the other same-aged embryos. The combination of persistent cell corpses and late-embryonic lethality was defined as a new phenotypical class [24], [36].

**Figure 6:**
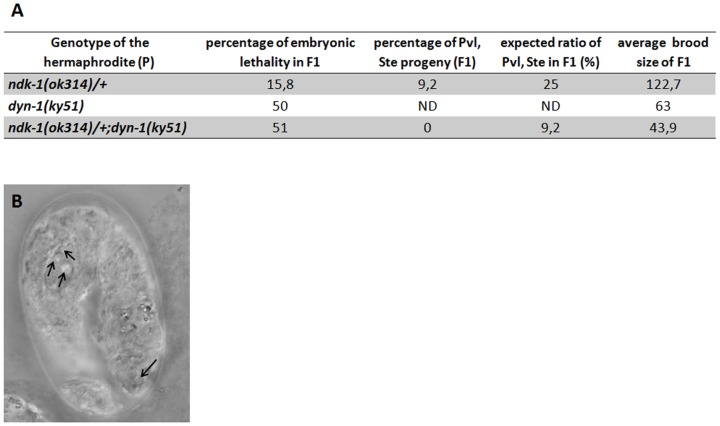
*ndk-1*(*ok314*);*dyn-1*(*ky51*) double mutants are lethal. **A**: In the F1 progeny of *ndk-1*(-)*/+* heterozygotes only 9.2% homozygous Pvl, Ste adults can be observed instead of the expected 25%, since 15.8% of *ndk-1*(*ok314*) homozygotes die as embryos at 25°C. At the restrictive temperature (25°C) 50% of *dyn-1*(*ky51*) single mutants die as embryos. At 25°C, in the F1 progeny of *dyn-1(ky51);ndk-1(ok314)/+* animals we got decreased brood size and we did not notice any Pvl, Ste animals, suggesting that the double mutants are not viable. **B**: 3-fold stage homozygous *ndk-1*(*ok314*) embryo shows late embryonic lethality with persistent cell corpses. Arrows indicate apoptotic corpses.

Previously we characterized Ndk-1 mutant phenotypes and showed that in the progeny of *ndk-1(ok314)/+* heterozygotes 12% of *ndk-1*(*ok314*) homozygotes die as embryos [25]. Further analysis revealed that these dying *ndk-1*(*ok314*) embryos also show the Dyn-1-like late embryonic lethality phenotype with persistent cell corpses (***Figure 6B***).

Next, to see whether *ndk*-1 and *dyn-1* also interact in the worm, we decided to generate *ndk-1*(-);*dyn-1*(-) double mutants. For crossing we used *ndk-1*(*ok314*) mutants and the viable *ky51* allele of *dyn-1*, wherein we took the advantage of thermosensitivity.

We performed this experiment at the restrictive temperature for *ky51* (25°C) (***Figure 6A***). 15.8% of *ndk-1*(*ok314*) homozygotes died as embryos and *ndk-1/+* heterozygotes laid on the average 122.7 eggs. We measured approximately 50% lethality of *dyn-1*(*ky51*) single mutants and 51% lethality in the F1 progeny of *dyn-1(ky51);ndk-1(ok314)/+* animals, but the broodsize was decreased (43.9) in the latter category compared to *dyn-1* single mutants (63). At the restrictive temperature we did not notice any Pvl, Ste (double mutants) in the F1 progeny. Putting the F1 animals to the permissive temperature (15°C) the Pvl, Ste phenotype appears in F2 generation. Therefore these observations suggest that the double mutant is not viable indicating that *ndk-1* and *dyn-1* interact genetically in the worm as well.

## Discussion

Group I NDPKs are negative regulators of cell migration and motility. To investigate the function of the sole worm group I homolog, NDK-1 in cell migration, we overexpressed NDK-1 and human NM23-H1 and H2 in MDA-MB231T, an invasive breast carcinoma cell line. We found that NDK-1, similar to its human counterparts, dramatically suppressed the migratory potential of MDA-MB231T cells. This result shows that NDK-1 bears an evolutionary conserved function in cell migration and that *C. elegans* serves as a tractable model system to monitor the effects of the NDPK gene family.

Next, we studied the role of *ndk-1* in the process of the migration of distal tip cells (DTCs) which are responsible for leading gonad morphogenesis. During larval development distal tip cells guide the migrating gonad arms which finally form two U-shaped tubes [16]. Studying different transgenic strains, we detected NDK-1::GFP expression in DTCs. Detailed analysis of *ndk-1* loss of function mutants showed different DTC migration defects, but predominantly, incompletely elongated gonad arms were the consistent feature. The majority of *ok314* mutants showed a third (dorsal) phase migratory defect, as a consequence of a premature stop of DTCs, after the turn, that was not present in wild-type worms.

Thus, overexpression of NDK-1 inhibited cell migration and reduced motility of metastatic breast adenocarcinoma cells, but loss of NDK-1 function in the worm predominantly caused incomplete gonadal migration and not excess migration. These data suggest that NDK-1 might have opposing functions depending on the cellular environment, e.g. NDK-1 might promote cell migration in the worm, but inhibit it in human cell lines. In support of this idea, recent work showed that NDPK affects *Dictyostelium* growth in opposing ways in different cellular environment [10]. To further investigate this issue, it will be important to overexpress NDK-1 in the worm and analyze the phenotypes in response to overexpression.

Previously *nm23* genes were linked to integrin signaling in human studies [28]–[30]. In *C. elegans*, integrins act in the dorsal/third phase of DTC migration [16], [21], where the majority of defects conferred by *ndk-1*(*ok314*) mutants are also observed. Consistently, NDK-1::GFP expression was detected in the third phase of migration (from L3 to adulthood). Based on these findings we performed an epistasis analysis with genes involved in CED-10/Rac signaling (*vab-3/Pax6, ina-1/alpha-integrin, unc-73/Trio, mig-2/RhoG, ced-5/Dock180, ced-12/Elmo, ced-10/Rac*) and with *abl-1/ABL* and *abi-1/ABI* which were shown to act in parallel to the CED-10 pathway. All mutants of CED-10 pathway genes have large gonads with extra turns. Putative null mutant alleles of *abl-1* (*ok171* and *n1963*) display no obvious gonad phenotype [22]. We observed 30% of abnormal gonad arms in *abi-1*(*ok640*) mutants in which the overshoot phenotype was the most frequent (14%). This result is consistent with previous data, e.g. *abi-1* mutations or *abi-1*(*RNAi*) results in overextensioned DTC migration [22]. Loss of *ndk-1* reduced the extra turn phenotype of *vab-3, ina-1, unc-73, mig-2*, *ced-5, ced-12* and *ced-10* mutants and mostly resulted in Ndk-1-like gonad (similar to that of *ndk-1*(-) mutants). These results lead to the hypothesis that NDK-1 might act downstream of or in parallel to *ced-10*. Although *abl-1*(*ok171*) was able to suppress the extra turn phenotype of the *ced* mutants [22], it did not influence the reduced migration of *ndk-1* mutants. Unlike *abl-1*(*ok171*), *abi-1*(*ok640*) mutation was able to restore partially the DTC migration defects of *ndk-1*(*ok314*) animals, showing a genetic interaction between *ndk-1* and *abi-1*.

Both distal tip cell migration and engulfment of apoptotic cell corpses require precise regulation of cytoskeleton to extend cell surfaces [19]–[20], [22]. Although *nm23* has been implicated in apoptosis [7], [39]–[43] in these studies NM23's function was detected in the dying, not in the engulfing cells. In *C. elegans*, during somatic cell death neighboring cells engulf the nascent apoptotic corpses; however germ cell corpses are eliminated by specialized engulfing cells, the sheath cells [17]. We noticed increased numbers of apoptotic corpses in the germline of *ndk-1*(*RNAi*) animals and in *ndk-1*(*ok314*) embryos, furthermore, NDK-1 is expressed in sheath cells (this study) as well as in embryos [25]. These results suggested that NDK-1 plays a role in engulfment of apoptotic cells. We note that subsequent experiments are necessary to further confirm the role of NDK-1 in engulfment, such as investigating the persistance of apoptotic corpses in *ndk-1*(-) single mutants.

Starting from the observation that *ndk-1* genetically interacts with *abi-1* in DTC migration and functions likely downstream of *ced-10*, we hypothesized that *ndk-1* regulates engulfment in the same manner. We analyzed *ndk-1(-), abi-1(-) and ced-10(-)* single mutant embryos and *ndk-1(-);abi-1(-) and ndk-1(-);ced-10(-)* double mutants. *ced-10*(-), *ndk-1*(-) and *abi-1*(-) mutants contain more apoptotic corpses than wild type animals. Moreover, we observed an increased number of corpses, an additive phenotype in *ndk-1*(-);*abi-1*(-) double mutant embryos, whereas *ndk-1*(-);*ced-10*(-) double mutants displayed apoptotic corpses reminiscent to *ced-10*(-) single mutants. These results suggest that *ndk-1* acts in parallel to *abi-1* and downstream of *ced-10* during apoptotic engulfment.

We also analyzed the putative interaction of *ndk-1* and *dyn-1/dynamin*, which is a downstream factor of the CED-1 pathway involved in engulfment. The CED-1 pathway is thought to recruit membranes to extend the surface of the engulfing cell, and DYN-1 is proposed to organize vesicle transport during this process. Interaction between NDPKs and Dynamins in endocytosis is well known in other model systems [9], [12]–[13], [36], [44]. *ndk-1*(-) and *dyn-1*(-) single mutants display a phenotypic similarity in the worm, the late-embryonic lethality with persistent cell corpses. Double mutant analysis revealed that *ndk-1(ok314);dyn-1(ky51)* animals are not viable indicating a genetic interaction between the two genes in *C. elegans* as well.

Together, we propose that NDK-1 acts downstream of CED-10 and in parallel to ABI-1 in the process of engulfment and DTC migration. In addition, *ndk-1* shows a genetic interaction with *dyn-1/dynamin*, which raises the possibility that NDK-1 might represent a converging point of the CED-1 and CED-10 pathways. Interestingly, in 10% of *ndk-1*(-),*ced-10*(-) double mutant embryos we observed an increased number of cell corpses (note that in 90% of the double mutants the corps number is similar to *ced-10*(-) single mutants). The convergence of the two pathways would explain this partial additivity observed in *ndk-1*(-);*ced-10*(-) double mutants. There are also data showing that silencing of *dyn-1* results in a DTC migration phenotype [38], although the CED-1 pathway is not considered to influence DTC migration. Earlier it was suggested that the CED-10 and CED-1 engulfment pathways converge at *ced-10* [45], but later studies dealing with DYN-1's function presume that the two engulfment pathways do not converge [36], [46]. Recently, it was found that the CED-1 pathway not only regulates membrane recruitment during engulfment, but is also connected through CHC-1/clathrin heavy chain to F-actin assembly [46]. Thus, the CED-1 pathway – besides the CED-10 pathway – is also linked to cytoskeleton remodeling. Earlier studies confirmed NDPKs' role in cell migration and endocytosis [12], [26], processes, which are both related also to CED-1 and CED-10 pathways.

In summary, our genetic analysis shows that *ndk-1* acts downstream of *ced-10* in the regulation of engulfment and DTC migration and genetically interacts with *dyn-1/dynamin*. These data might contribute to our understanding of how NDK-1 exerts its function in the rearrangement of the cytoskeleton. Although NDPKs were already related to phagocytosis in different systems [10], [47], this is the first time that we link NDK-1/NDPK to apoptotic engulfment. We show that impaired NDK-1 function causes inefficient engulfment. In mammals it is known that failure of engulfment leads to inflammation [48], moreover inflammation favors tumour progression in certain circumstances [49]. In further studies it would be interesting to overexpress or silence NDK-1 in specialized engulfing cells, such as the macrophage-monocyte system, to further investigate NDPKs' function in apoptotic engulfment.

## Materials and Methods

### Nematode strains and alleles

*C. elegans* strains were cultivated at 20°C on NGM plates seeded with OP50 bacteria [50]. The N2 Bristol strain was used as the wild-type strain. The following transgenic or mutant strains were used:

TTV1 ndk-1(ok314) I/hT2[bli-4(e937) let-?(q782) qIs48](I;III); TTV2 eluEx1[NDK-1::gfp; unc-119(+)];unc-119(ed3)III; TTV3 eluSi1[NDK-1::GFP+cb-unc-119(+)]III; LGI: MT11068 ced-12(n3261), CB3203 ced-1(e1735), CB936 unc-73(e936), LGIII: RB829 B0336.6(ok640) corresponds to abi-1(ok640), NG39 ina-1(gm39), LGIV: MT4434 ced-5(n1812), MT1522 ced-3(n717), MT5013 ced-10(n1993), LGV: MD701 bcIs39[P(lim-7)ced-1::GFP+lin-15(+)], LGX: CX51 dyn-1(ky51), CB3304 vab-3(e1796), LE815 mig-2(lg13), XR1 abl-1(ok171).

Information about alleles can be found at www.wormbase.org.

### RNA interference

For RNAi through ingested dsRNAs, an XhoI digested fragment of the full-length cDNA corresponding to F25H2.5 (*yk1105e04*) was cloned into pPD129.36, and the obtained construct was transformed into HT115 (DE3) bacteria. RNAi experiments were performed at 25 °C essentially as described [51].

#### Quantitation of DTC defects

Adult animals were anaesthetized and examined visually. Only completely visible gonads were analyzed. Gonadal length was defined as reduced when the gonad tip was before the vulva and as overshoot when the gonad was elongated past vulva. Extra turn category means that we observed more than one complete turn. DTC migration was scored as ‘other’ when the gonad was morphologically abnormal (wrong direction, lack of the turn, wondering, extra arm, bizarre twists). As animals affected by *ndk-1*(*RNAi*), sterile adults possessing a protruding vulva were picked. For statistical analysis DTC migration phenotypes were grouped into three classes: reduced migration, migration positioned to vulva and excess migration (in the last class overshoot and extra turn categories were merged, see ***Figure 3 A***). Animals belonging to the ‘other’ category were not included in the statistical analysis, as they display a broad phenotypic variance, cannot be grouped in any category, and their ratio does not reach 10% in any mutant population used. *p*-values of pairwise comparisons of DTC defect rates were calculated by Fisher's exact probability test.

#### Quantitation of apoptotic cell corpses

Apoptotic corpses (as refractile discs) were scored in comma stage embryos directly by microscopy [33], using Nomarski/DIC (differential interference contrast) optics on Olympus BX51. *p*-values for pairwise comparisons of apoptotic cell corpse numbers were calculated using the Student's t-test.

### Cloning of the FLAG::NDK-1 (pcDNA3FLAG-nm23-NDK) construct

The insert of the construct was generated from the cDNA yk1105e04 by PCR using the following primers: 5′-cag aag atc tat gga cta caa gga cga cga cga taa gat gag caa cac tga gag aac c-3′ (which contains the flag sequence) and 5′-ata gtt tag cgg ccg ctt tat tcg tag acc cat gag ttg-3′. The insert was ligated into pCDNA3.1 using *BamHI* and *NotI* sites.

### Preparation of stably transfected MDA-MB-231T clones

MDA-MB-231T cells were transfected as follows: 4×10^6^ cells in DMEM supplemented with 10% FBS were subjected to electroporation (250 V, 950 μF) with 25 μg of each of the following plasmids: pcDNA3FLAG-*nm23*-NDK, pcDNA3FLAG-*nm23-H1*, pcDNA3MYC-*nm23-H2* and pcDNA3 as control. Post transfection the cells were seeded on 4×100 mm plates and incubated in DMEM supplemented with 1 mg/ml geneticin (Sigma) until development of resistant colonies. Positive clones were screened by Western blotting, propagated and stored in liquid nitrogen until further usage.

### Western blotting

For detection of stably transfected MDA-MB-231T clones 10^6^ cells of each clone was collected, washed in PBS and sonicated.

The samples (10 μg) were loaded on SDS-PAGE and electrotransfered to an Imobilon-PSQ membrane (Milipore). For detection of FLAG/NDK and FLAG/H1 the anti-FLAG M2 antibody (Sigma) was used while MYC/H2 was detected using anti-MYC antibody (Santa Cruz Biotechnology). Anti-α-tubulin antibody (Calbiochem) was used as loading control. After application of appropriate secondary antibodies the protein bands were visualized using Western Lightning Plus-ECL (PerkinElmer, Inc.). The image was acquired by Alliance 4.7 (Uvitec) and assembled in Adobe Photoshop.

### Cell line and migration assay

For migration assay metastatic human breast cancer MDA-MB-231T cells were used (a kind donation of Patricia S. Steeg, [52]. The cells were cultured in Dulbecco's modified Eagle medium (DMEM, Invitrogene) supplemented with 10% fetal bovine serum (FBS, Invitrogene), 2 mM glutamine, 100 U/mL penicillin and 100 μg/mL streptomycin in humidified chamber with 5% CO_2_ at 37°C.

For migration assay 3×10^5^ cells were seeded on 60 mm Petri dishes in DMEM supplemented with 10% fetal bovine serum. After 24 hours the cells were starved in serum free medium for another 24 hours. The cells were detached with 1 mM EDTA, centrifuged and resuspended in DMEM supplemented with 0,1% BSA. 2×10^4^ cells were placed into the upper chamber of Cell Culture Inserts (Beckton-Dickinson) and allowed to settle down for 20 minutes. DMEM supplemented with 1% FBS served as a chemoattractant and was added to the lower chamber. The cells were allowed to migrate for 5 hours, after which the medium was removed, non-migratory cells were removed from upper chamber with cotton swabs while bottom of the membranes with migrated cells were washed twice in PBS, and fixed in 4% formaldehyde for 15 minutes at room temperature. The cells were stained with 0.1% crystal violet, cut out from the inserts, mounted in (DAKO) on slide, analyzed by light microscopy, and photographed. The cells from four representative images of every clone were assembled in Adobe Photoshop and counted. The experiments were performed three times.

## Supporting Information

Figure S1 **Monitoring apoptotic corpses in embryos slightly after the comma stage in different mutant backgrounds.** **A–F**: Monitoring apoptotic corpses in wild-type embryos (**A**), *ndk-1*(*-*) (**D**), *abi-1*(*-*) (**B**), *ced-10*(*-*) (**C**) single mutants and *ndk-1*(*-*);*abi-1*(*-*) (**E**), *ndk-1*(*-*);*ced-10*(*-*) (**F**) double mutants using DIC optics. Embryos slightly after the comma stage were scored. Each panel shows two focal planes (**A–F**). Arrowheads indicate apoptotic corpses.
(TIF)

## References

[1] Marino N, Marshall JC, Steeg PS (2011) Protein-protein interactions: a mechanism regulating the anti-metastatic properties of Nm23-H1. Naunyn Schmiedebergs Arch Pharmacol 384: 351–62. 21713383 10.1007/s00210-011-0646-6 PMC6545597

[2] Desvignes T, Pontarotti P, Fauvel C, Bobe J (2009) Nme protein family evolutionary history, a vertebrate perspective. BMC Evol Biol 9: 256. 19852809 10.1186/1471-2148-9-256 PMC2777172

[3] Besant PG, Tan E, Attwood PV (2003) Mammalian protein histidine kinases. Int J Biochem Cell Biol 35: 297–309. 12531242 10.1016/s1357-2725(02)00257-1

[4] Steeg PS, Palmieri D, Ouatas T, Salerno M (2003) Histidine kinases and histidine phosphorylated proteins in mammalian cell biology, signal transduction and cancer. Cancer Lett 190: 1–12. 12536071 10.1016/s0304-3835(02)00499-8

[5] Ma D, McCorkle JR, Kaetzel DM (2004) The metastasis suppressor NM23-H1 possesses 3′-5′ exonuclease activity. J Biol Chem 279: 18073–84. 14960567 10.1074/jbc.M400185200

[6] Zhang Q, McCorkle JR, Novak M, Yang M, Kaetzel DM (2011) Metastasis suppressor function of NM23-H1 requires its 3′-5′ exonuclease activity. Int J Cancer 128: 40–50. 20209495 10.1002/ijc.25307 PMC2946830

[7] Fan Z, Beresford PJ, Oh DY, Zhang D, Lieberman J (2003) Tumor suppressor NM23-H1 is a granzyme A-activated DNase during CTL-mediated apoptosis, and the nucleosome assembly protein SET is its inhibitor. Cell 112: 659–72. 12628186 10.1016/s0092-8674(03)00150-8

[8] Postel EH, Weiss VH, Beneken J, Kirtane A (1996) Mutational analysis of NM23-H2/NDP kinase identifies the structural domains critical to recognition of a c-*myc* regulatory element. Proc Natl Acad Sci USA 93: 6892–7. 8692914 10.1073/pnas.93.14.6892 PMC38904

[9] Nallamothu G, Dammai V, Hsu T (2009) Developmental function of *Nm23/awd*: a mediator of endocytosis. Mol Cell Biochem 329: 35–44. 19373545 10.1007/s11010-009-0112-7 PMC2721904

[10] Annesley SJ, Bago R, Bosnar MH, Filic V, Marinović M, et al (2011) *Dictyostelium discoideum* nucleoside diphosphate kinase C plays a negative regulatory role in phagocytosis, macropinocytosis and exocytosis. PLoS One. 6: e26024. 21991393 10.1371/journal.pone.0026024 PMC3186806

[11] Nallamothu G, Woolworth JA, Dammai V, Hsu T (2008) *awd*, the homolog of metastasis suppressor gene *Nm23*, regulates *Drosophila* epithelial cell invasion. Mol Cell Biol 28: 1964–73. 18212059 10.1128/MCB.01743-07 PMC2268403

[12] Dammai V, Adryan B, Lavenburg KR, Hsu T (2003) *Drosophila awd*, the homolog of human *nm23*, regulates FGF receptor levels and functions synergistically with *shi/dynamin* during tracheal development. *Genes & Dev* 17: 2812–2824. 14630942 10.1101/gad.1096903 PMC280629

[13] Baillat G, Gaillard S, Castets F, Monneron A (2002) Interactions of phocein with nucleoside-diphosphate kinase, Eps15, and Dynamin I. J Biol Chem 277: 18961–6. 11872741 10.1074/jbc.M108818200

[14] Blelloch R, Newman C, Kimble J (1999) Control of cell migration during *Caenorhabditis elegans* development. Curr Opin Cell Biol 11: 608–13. 10508660 10.1016/s0955-0674(99)00028-9

[15] Montell DJ (1999) The genetics of cell migration in *Drosophila melanogaster* and *Caenorhabditis elegans* development. Development 126: 3035–46. 10375496 10.1242/dev.126.14.3035

[16] Lehmann R (2001) Cell migration in invertebrates: clues from border and distal tip cells. Curr Opin Genet Dev 11: 457–63. 11448633 10.1016/s0959-437x(00)00217-3

[17] Gumienny TL, Lambie E, Hartwieg E, Horvitz HR, Hengartner MO (1999) Genetic control of programmed cell death in the *Caenorhabditis elegans* hermaphrodite germline. Development 126: 1011–1022. 9927601 10.1242/dev.126.5.1011

[18] Gartner A, Boag PR, Blackwell TK (2008) Germline Survival and Apoptosis. WormBook. 10.1895/wormbook.1.145.1 PMC4781258 18781708

[19] Wu YC, Horvitz HR (1998) *C. elegans* phagocytosis and cell-migration protein CED-5 is similar to human DOCK180. Nature 392: 501–4. 9548255 10.1038/33163

[20] Reddien PW, Horvitz HR (2000) CED-2/CrkII and CED-10/Rac control phagocytosis and cell migration in *Caenorhabditis elegans* . Nat Cell Biol 2: 131–6. 10707082 10.1038/35004000

[21] Meighan CM, Schwarzbauer JE (2007) Control of *C. elegans* hermaphrodite gonad size and shape by *vab-3*/Pax6-mediated regulation of integrin receptors. Genes Dev 21: 1615–20. 17606640 10.1101/gad.1534807 PMC1899471

[22] Hurwitz ME, Vanderzalm PJ, Bloom L, Goldman J, Garriga G, et al (2009) Abl kinase inhibits the engulfment of apoptotic cells in Caenorhabditis elegans. PLoS Biol 7: e99. 19402756 10.1371/journal.pbio.1000099 PMC2672617

[23] Hsu TY, Wu YC (2010) Engulfment of apoptotic cells in *C. elegans* is mediated by integrin α/SRC signaling. Curr Biol 20: 477–86. 20226672 10.1016/j.cub.2010.01.062

[24] Mangahas PM, Zhou Z (2005) Clearance of apoptotic cells in *Caenorhabditis elegans* . Semin Cell Dev Biol 16: 295–306. 15797839 10.1016/j.semcdb.2004.12.005

[25] Masoudi N, Fancsalszky L, Pourkarimi E, Vellai T, Alexa A, et al (2013) The NM23-H1/H2 homolog NDK-1 is required for full activation of Ras signaling in *C. elegans* . Development 140: 3486–95. 23900546 10.1242/dev.094011 PMC3737725

[26] Boissan M, De Wever O, Lizarraga F, Wendum D, Poincloux R, et al (2010) Implication of metastasis suppressor NM23-H1 in maintaining adherens junctions and limiting the invasive potential of human cancer cells. Cancer Res 70: 7710–22. 20841469 10.1158/0008-5472.CAN-10-1887

[27] Perina D, Bosnar MH, Bago R, Mikoč A, Harcet M, et al (2011) Sponge non-metastatic Group I Nme gene/protein - structure and function is conserved from sponges to humans. BMC Evol Biol 11: 87. 21457554 10.1186/1471-2148-11-87 PMC3078890

[28] Fournier HN, Albiges-Rizo C, Block MR (2003) New insights into Nm23 control of cell adhesion and migration. J Bioenerg Biomembr 35: 81–87. 12848345 10.1023/a:1023450008347

[29] Miyamoto M, Iwashita S, Yamaguchi S, Ono Y (2009) Role of nm23 in the regulation of cell shape and migration via Rho family GTPase signals. Mol Cell Biochem 329: 175–9. 19381785 10.1007/s11010-009-0106-5

[30] She S, Xu B, He M, Lan X, Wang Q (2010) Nm23-H1 suppresses hepatocarcinoma cell adhesion and migration on fibronectin by modulating glycosylation of integrin beta1. J Exp Clin Cancer Res 29: 93. 20618991 10.1186/1756-9966-29-93 PMC2909969

[31] Lee M, Cram EJ, Shen B, Schwarzbauer JE (2001) Roles for βpat-3 integrins in development and function of *Caenorhabditis elegans* muscles and gonads. J Biol Chem 276: 36404–10. 11473126 10.1074/jbc.M105795200

[32] Lee M, Shen B, Schwarzbauer JE, Ahn J, Kwon J (2005) Connections between integrins and Rac GTPase pathways control gonad formation and function in *C. elegans* . Biochim Biophys Acta. 1723: 248–55. 15716039 10.1016/j.bbagen.2005.01.003

[33] Sulston JE, Horvitz HR (1977) Post-embryonic cell lineages of the nematode, *Caenorhabditis elegans* . Dev Biol 56: 110–156. 838129 10.1016/0012-1606(77)90158-0

[34] Sulston JE, Schierenberg E, White JG, Thomson N (1983) The embryonic cell lineage of the nematode *Caenorhabditis elegans* . Dev Biol 100: 64–119. 6684600 10.1016/0012-1606(83)90201-4

[35] Lundquist EA, Reddien PW, Hartwieg E, Horvitz HR, Bargmann CI (2001) Three *C. elegans* Rac proteins and several alternative Rac regulators control axon guidance, cell migration and apoptotic cell phagocytosis. Development 128: 4475–88. 11714673 10.1242/dev.128.22.4475

[36] Yu X, Odera S, Chuang CH, Lu N, Zhou Z (2006) *C. elegans* Dynamin mediates the signaling of phagocytic receptor CED-1 for the engulfment and degradation of apoptotic cells. Dev Cell 10: 743–57. 16740477 10.1016/j.devcel.2006.04.007

[37] Woolworth JA, Nallamothu G, Hsu T (2009) The *Drosophila* metastasis suppressor gene *Nm23* homolog, *awd*, regulates epithelial integrity during oogenesis. Mol Cell Biol 29: 4679–90. 19581292 10.1128/MCB.00297-09 PMC2725718

[38] Cram EJ, Shang H, Schwarzbauer JE (2006) A systematic RNA interference screen reveals a cell migration gene network in *C. elegans* . J Cell Sci 119: 4811–8. 17090602 10.1242/jcs.03274

[39] Venturelli D, Martinez R, Melotti P, Casella I, Peschle C, et al (1995) Overexpression of DR-nm23, a protein encoded by a member of the nm23 gene family, inhibits granulocyte differentiation and induces apoptosis in 32Dc13 myeloid cells. Proc Natl Acad Sci USA 92: 7435–9. 7638209 10.1073/pnas.92.16.7435 PMC41354

[40] Volm M, Mattern J, Koomägi R (1998) Association between nm23-H1 expression, proliferation and apoptosis in non-small cell lung carcinomas. Clin Exp Metastasis 16: 595–602. 9932606 10.1023/a:1006588601683

[41] Negroni A, Venturelli D, Tanno B, Amendola R, Ransac S, et al (2000) Neuroblastoma specific effects of DR-nm23 and its mutant forms on differentiation and apoptosis. Cell Death Differ 7: 843–50. 11042679 10.1038/sj.cdd.4400720

[42] Kang Y, Lee DC, Han J, Yoon S, Won M, et al (2007) NM23-H2 involves in negative regulation of Diva and Bcl2L10 in apoptosis signaling. Biochem Biophys Res Commun 359: 76–82. 17532299 10.1016/j.bbrc.2007.05.090

[43] Choudhuri T, Murakami M, Kaul R, Sahu SK, Mohanty S, et al (2010) Nm23-H1 can induce cell cycle arrest and apoptosis in B cells. Cancer Biol Ther 9: 1065–78. 20448457 10.4161/cbt.9.12.11995 PMC5965688

[44] Krishnan KS, Rikhy R, Rao S, Shivalkar M, Mosko M, et al (2001) Nucleoside diphosphate kinase, a source of GTP, is required for dynamin-dependent synaptic vesicle recycling. Neuron 30: 197–210. 11343655 10.1016/s0896-6273(01)00273-2

[45] Kinchen JM, Cabello J, Klingele D, Wong K, Feichtinger R, et al (2005) Two pathways converge at CED-10 to mediate actin rearrangement and corpse removal in *C. elegans* . Nature 434: 93–9. 15744306 10.1038/nature03263

[46] Shen Q, He B, Lu N, Conradt B, Grant BD, et al (2013) Phagocytic receptor signaling regulates clathrin and epsin-mediated cytoskeletal remodeling during apoptotic cell engulfment in *C. elegans* . Development 140: 3230–43. 23861060 10.1242/dev.093732 PMC3931732

[47] Sun J, Wang X, Lau A, Liao TY, Bucci C, et al (2010) Mycobacterial nucleoside diphosphate kinase blocks phagosome maturation in murine RAW 264.7 macrophages. PLoS One 5: e8769. 20098737 10.1371/journal.pone.0008769 PMC2808246

[48] Elliott MR, Ravichandran KS (2010) Clearance of apoptotic cells: implications in health and disease. J Cell Biol 189: 1059–1070. 20584912 10.1083/jcb.201004096 PMC2894449

[49] Hanahan D, Weinberg RA (2011) Hallmarks of cancer: the next generation. Cell 2011 144: 646–74. 10.1016/j.cell.2011.02.013 21376230

[50] Brenner S (1974) The genetics of *Caenorhabditis elegans* . Genetics 77: 71–94. 4366476 10.1093/genetics/77.1.71 PMC1213120

[51] Timmons L (2006) Construction of plasmids for RNA interference and in vitro transcription of double-stranded RNA. Methods Mol Biol 351: 109–17. 16988429 10.1385/1-59745-151-7:109

[52] Palmieri D, Halverson DO, Ouatas J, Horak CE, Salerno M, et al (2005) Medroxyprogesterone acetate elevation of Nm23-H1 metastasis suppressor expression in hormone receptor-negative breast cancer. J Natl Cancer Inst 97: 632–42. 15870434 10.1093/jnci/dji111

