# Supplementary figures and images for: NDK-1, the Homolog of NM23-H1/H2 Regulates Cell Migration and Apoptotic Engulfment in C. elegans
Source: PLoS One. 2014 Mar 21;9(3):e92687. doi: 10.1371/journal.pone.0092687 (PMC3962447; doi:10.1371/journal.pone.0092687)

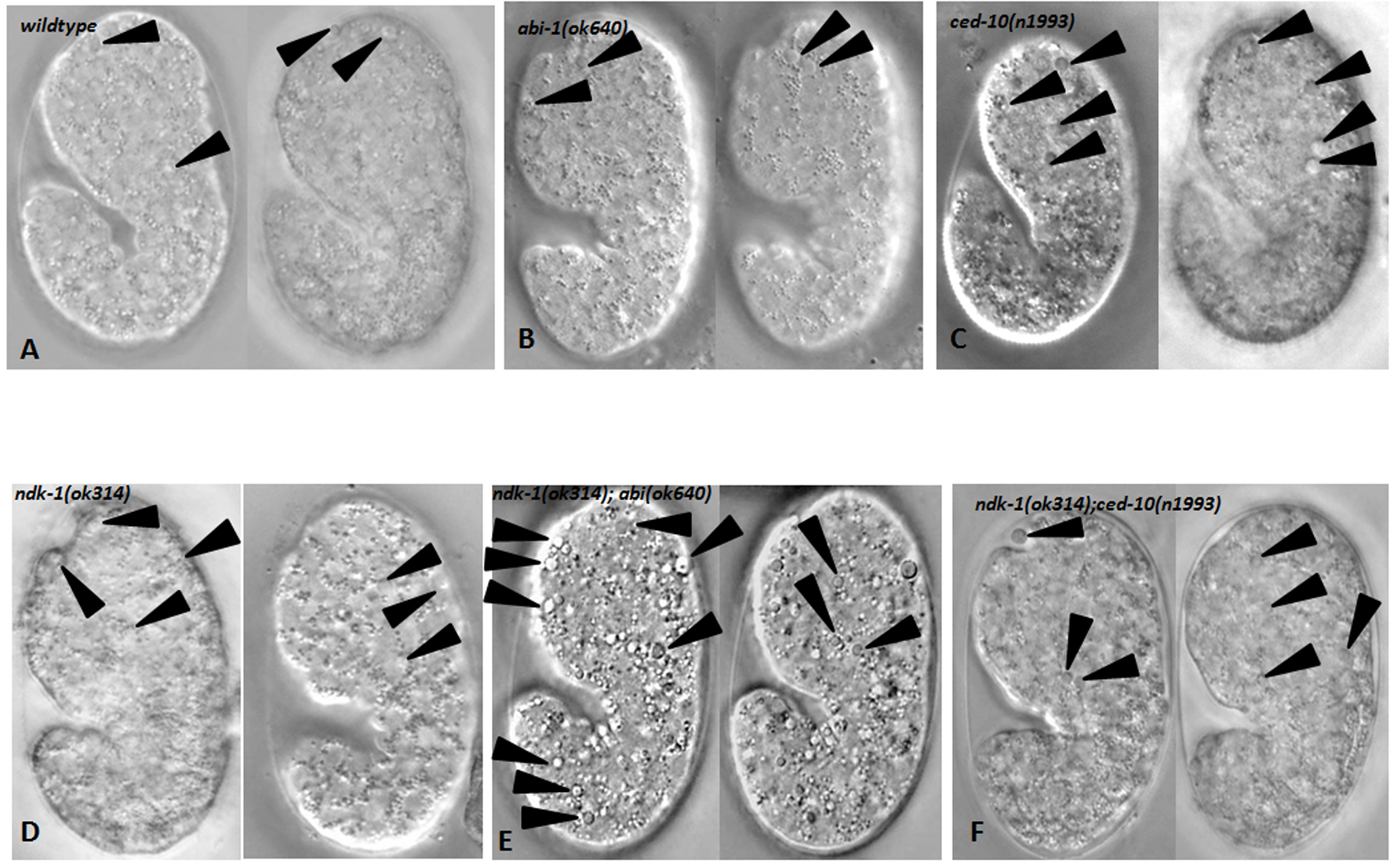

Supplement: Figure S1 — Monitoring apoptotic corpses in embryos slightly after the comma stage in different mutant backgrounds. A–F: Monitoring apoptotic corpses in wild-type embryos (A), ndk-1(-) (D), abi-1(-) (B), ced-10(-) (C) single mutants and ndk-1(-);abi-1(-) (E), ndk-1(-);ced-10(-) (F) double mutants using DIC optics. Embryos slightly after the comma stage were scored. Each panel shows two focal planes (A–F). Arrowheads indicate apoptotic corpses. (TIF) [file pone.0092687.s001.tif]
